# Assessment of SARS-CoV-2 Screening Strategies to Permit the Safe Reopening of College Campuses in the United States

**DOI:** 10.1001/jamanetworkopen.2020.16818

**Published:** 2020-07-31

**Authors:** A. David Paltiel, Amy Zheng, Rochelle P. Walensky

**Affiliations:** 1 Public Health Modeling Unit, Yale School of Public Health, New Haven, Connecticut; 2 Harvard Medical School, Boston, Massachusetts; 3 Medical Practice Evaluation Center, Division of Infectious Diseases, Massachusetts General Hospital, Boston

## Abstract

**Importance:**

The coronavirus disease 2019 (COVID-19) pandemic poses an existential threat to many US residential colleges; either they open their doors to students in September or they risk serious financial consequences.

**Objective:**

To define severe acute respiratory syndrome coronavirus 2 (SARS-CoV-2) screening performance standards that would permit the safe return of students to US residential college campuses for the fall 2020 semester.

**Design, Setting, and Participants:**

This analytic modeling study included a hypothetical cohort of 4990 students without SARS-CoV-2 infection and 10 with undetected, asymptomatic SARS-CoV-2 infection at the start of the semester. The decision and cost-effectiveness analyses were linked to a compartmental epidemic model to evaluate symptom-based screening and tests of varying frequency (ie, every 1, 2, 3, and 7 days), sensitivity (ie, 70%-99%), specificity (ie, 98%-99.7%), and cost (ie, $10/test-$50/test). Reproductive numbers (R_t_) were 1.5, 2.5, and 3.5, defining 3 epidemic scenarios, with additional infections imported via exogenous shocks. The model assumed a symptomatic case fatality risk of 0.05% and a 30% probability that infection would eventually lead to observable COVID-19–defining symptoms in the cohort. Model projections were for an 80-day, abbreviated fall 2020 semester. This study adhered to US government guidance for parameterization data.

**Main Outcomes and Measures:**

Cumulative tests, infections, and costs; daily isolation dormitory census; incremental cost-effectiveness; and budget impact.

**Results:**

At the start of the semester, the hypothetical cohort of 5000 students included 4990 (99.8%) with no SARS-CoV-2 infection and 10 (0.2%) with SARS-CoV-2 infection. Assuming an R_t_ of 2.5 and daily screening with 70% sensitivity, a test with 98% specificity yielded 162 cumulative student infections and a mean isolation dormitory daily census of 116, with 21 students (18%) with true-positive results. Screening every 2 days resulted in 243 cumulative infections and a mean daily isolation census of 76, with 28 students (37%) with true-positive results. Screening every 7 days resulted in 1840 cumulative infections and a mean daily isolation census of 121 students, with 108 students (90%) with true-positive results. Across all scenarios, test frequency was more strongly associated with cumulative infection than test sensitivity. This model did not identify symptom-based screening alone as sufficient to contain an outbreak under any of the scenarios we considered. Cost-effectiveness analysis selected screening with a test with 70% sensitivity every 2, 1, or 7 days as the preferred strategy for an R_t_ of 2.5, 3.5, or 1.5, respectively, implying screening costs of $470, $910, or $120, respectively, per student per semester.

**Conclusions and Relevance:**

In this analytic modeling study, screening every 2 days using a rapid, inexpensive, and even poorly sensitive (>70%) test, coupled with strict behavioral interventions to keep R_t_ less than 2.5, is estimated to maintain a controllable number of COVID-19 infections and permit the safe return of students to campus.

## Key Points

**Question:**

What screening and isolation programs for severe acute respiratory syndrome coronavirus 2 (SARS-CoV-2) will keep students at US residential colleges safe and permit the reopening of campuses?

**Findings:**

This analytic modeling study of a hypothetical cohort of 4990 college-age students without SARS-CoV-2 infection and 10 students with undetected asymptomatic cases of SARS-CoV-2 infection suggested that frequent screening (every 2 days) of all students with a low-sensitivity, high-specificity test might be required to control outbreaks with manageable isolation dormitory utilization at a justifiable cost.

**Meaning:**

In this modeling study, symptom-based screening alone was not sufficient to contain an outbreak, and the safe reopening of campuses in fall 2020 may require screening every 2 days, uncompromising vigilance, and continuous attention to good prevention practices.

## Teaser

This analytic modeling study defines the screening performance standards for severe acute respiratory syndrome coronavirus 2 (SARS-CoV-2) tests that would permit the safe return of students to US residential college campuses for the fall 2020 semester.

## Introduction

Universities across the United States are struggling with the question of whether and how to reopen for the fall 2020 semester.^1,2^ Residential colleges, with communal living arrangements, shared dining spaces, intimate classrooms, and a population of young adults anxious to socialize, pose a particular challenge. In the absence of an effective vaccine, a proven therapy, and/or sufficient herd immunity, the best hope for reopening campuses in the fall is likely to be a robust strategy of behavior-based prevention combined with regular monitoring to rapidly detect, isolate, and contain new severe acute respiratory syndrome coronavirus 2 (SARS-CoV-2) infections when they occur.^3^

Evidence on the available monitoring technologies and their performance is limited and rapidly evolving. The US Food and Drug Administration is currently evaluating more than 100 candidate tests that screen for the presence of SARS-CoV-2 infection or antibodies.^4^ There are many uncertainties, including the logistics of deployment; the ease and comfort of sample collection; and the accuracy, scalability, turnaround time, and cost of test kits. After a new coronavirus disease 2019 (COVID-19) case is detected, further questions emerge regarding how to conduct subsequent tracing; how to isolate detected cases in the context of congregate housing arrangements; and how to protect other at-risk populations, including faculty, staff, and members of the surrounding community.^5^ These uncertainties underscore the pressing need for both a generalized assessment of population-wide screening for SARS-CoV-2 and a comprehensive plan for reopening universities.

For many US colleges, COVID-19 poses an existential threat: either they open their doors to students in September or they suffer severe financial consequences.^6^ University administrators struggling with this dilemma must nevertheless keep in mind that their first priority is the safety of the students in their care. We offer specific recommendations on the design of a virologic monitoring program that will keep students safe at an affordable cost. Our specific research objectives were, first, to define the minimum performance attributes of a SARS-CoV-2 monitoring program (eg, frequency, sensitivity, specificity, and cost) that could ensure that college students are kept safe; second, to understand how those minimum performance standards might change under varying assumptions about the severity of the epidemic and the success of behavioral and social distancing interventions; third, to suggest what isolation and treatment capacity would need to be in place; and fourth, to forecast what testing might cost and to help decision-makers understand that information to address the question of a screening and monitoring program’s value.

## Methods

### Study Design

We adapted a simple compartmental epidemic model to capture the essential features of the situation facing university decision-makers that included the epidemiology of SARS-CoV-2; the natural history of COVID-19 illness; and regular mass screening to detect, isolate, and contain the presence of SARS-CoV-2 in a residential college setting (eFigure 1 in the Supplement). A spreadsheet implementation of the model permitted us to vary critical epidemic parameters and to examine how different test performance attributes (ie, frequency, sensitivity, specificity, and cost) would translate to outcomes. Model input data (Table 1)^7,8,9,10,11,12,13,14,15,16,17,18,19^ were obtained from a variety of published sources, adhering whenever possible to the data guidance for modelers recently issued by the US Centers for Disease Control and Prevention and the Office of the Assistant Secretary for Preparedness and Response. We defined 3 increasingly pessimistic epidemic scenarios and estimated both cumulative outcomes (eg, tests administered, number of true-positive and false-positive results, number of new infections, and person-days requiring isolation) and economic performance (eg, cost, incremental cost-effectiveness, and budget impact) during an abbreviated 80-day semester, running from Labor Day through Thanksgiving.^2^ We assumed a medium-sized college setting with a target population of 5000 students, all of them younger than 30 years and nonimmune, living in a congregate setting.^19,20^ We seeded this population with 10 undetected, asymptomatic cases of SARS-CoV-2 infection. A publicly accessible version of the model implementation is available online.

**Table 1.** Model Input Parameters and Scenarios

Model parameter	Input	References
Compartments in initial population, No.		
Noninfected, susceptible	4990	US News and World Report,^19^ 2020
Infected, asymptomatic	10	Assumption
All other compartments	0	Assumption
Time horizon, d	80	Hubler,^2^ 2020
Disease dynamics		
Mean incubation time, θ	3 d	He et al,^8^ 2020
Time to recovery, 1/ρ	14 d	Lauer et al,^10^ 2020; CDC,^11^ 2020
Time to false-positive return, 1/μ	1 d	Assumption
Probability of symptoms given infection, %	30	Day,^12^ 2020; Yang et al,^13^ 2020; Ing et al,^14^ 2020
Symptomatic case fatality ratio, %	0.05	CDC,^7^ 2020
Transmission rate, β	Dependent on R_t_	NA
Rate of symptom development, σ	Dependent on R_t_	NA
Scenarios		
Effective R_t_		
Best	1.5	CDC,^7^ 2020; Pitzer et al,^15^ 2020; Li et al,^16^ 2020
Base	2.5	
Worst	3.5	
Test specificity, ie, true-negative rate, %		
Best	99.7	Lieberman et al^17^ 2020; Zhen et al,^18^ 2020
Base	98.0	
Worst	98.0	
Exogenous infections per wk, No.		
Best	5	Assumption
Base	10	
Worst	25	
Test characteristics		
Sensitivity, ie, true-positive rate, %		
Test I	70	Assumption
Test II	80	
Test III	90	
Cost per test, $		
Test I	10	Assumption
Test II	25	
Test III	50	
Time to test result return, h	8	Assumption
Confirmatory test		
Sensitivity, %	100	Assumption
Cost, $	100	Assumption

Abbreviations: CDC, US Centers for Disease Control and Prevention; NA, not applicable; R_t_, reproduction number.

This analysis adheres to the Consolidated Health Economic Evaluation Reporting Standards (CHEERS) reporting guideline, where applicable. Because this study used only aggregate, published data, the institutional review boards of both the Massachusetts General Hospital and the Yale School of Medicine determined that this research did not involve human participants and did not require their review or approval.

### Compartmental Model

To the basic susceptible-exposed-infected-removed compartmental modeling framework, we added the following: the availability of regular, repeated screening with a test of imperfect sensitivity and specificity; the creation of a new compartment for uninfected persons receiving a false-positive test result; separation of the infected compartment to distinguish between asymptomatic patients with undetected infection, asymptomatic patients with detected infection (ie, true-positives), and observed symptomatic patients; and the importation of additional new infections via exogenous shocks (eg, infections transmitted to students by university employees or members of the surrounding community or during superspreader events, such as parties).

We defined 3 epidemic severity scenarios: a base case with a reproduction number (R_t_) of 2.5, test specificity of 98%, and the exogenous introduction of 10 new, undetected infections to the susceptible population each week; a worst case with an R_t_ of 3.5, test specificity of 98%, and 25 exogenous new infections every week; and a best case with an R_t _of 1.5, test specificity of 99.7%, and 5 exogenous new infections each week.

### Isolation

We assumed that after a lag of 8 hours, individuals receiving a positive test result (true or false) and those exhibiting COVID-19 symptoms would be moved from the general population to an isolation dormitory, where their infection would be confirmed, where they would receive supportive care, and from which no further transmissions would occur. The lag reflected both test turnaround delays and the time required to locate and isolate identified cases. Students with confirmed (ie, true-positive) results would remain in the isolation dormitory a mean of 14 days to ensure they were not infectious before proceeding to a recovered or immune state.^10,11^ Students with false-positive results would remain isolated for 24 hours, reflecting our assumption that a highly specific confirmatory test could overturn the original diagnosis, permitting them to return to the campus population.

We assumed a mean time from exposure to both infectiousness and screening detectability of 3 days, a symptomatic case fatality risk of 0.05%, and a 30% probability that infection would eventually lead to observable COVID-19–defining symptoms in this young cohort.^7,8,9,12,13,14^

### Screening

We sought to evaluate both existing SARS-CoV-2 detection methods and newer technologies that could plausibly be available in the near future. Accordingly, we considered a range of different test sensitivities (ie, 70%-99%), specificities (ie, 98%-99.7%),^17,18^ and per-test costs (ie, $10-$50). For each combination of these test characteristics, we considered both symptom-based screening and routine testing every 1, 2, 3, and 7 days. We assumed that a confirmatory test with 100% specificity could distinguish false-positive from true-positive results at a cost of $100.

### Cost-effectiveness

Next, we estimated incremental cost-effectiveness ratios, denominated in screening costs per infection averted. This measure of return on investment in screening was compared with a crude benchmark of value estimated using the following 4 terms: (1) COVID-related mortality (0.05% in persons of college age; 0.4% overall)^7^; (2) survival loss of 60 years per college-age fatality; 20 years overall^21^; (3) societal willingness-to-pay (WTP) threshold of $100 000 per year of life gained^22^; and (4) R_t_ + 1, assuming that each infection averted prevents half the R_t_ secondary infections among college-age students and half among other adult members of the campus community.^7,15,16^ This method yielded a maximum WTP to avert 1 infection of $5500 in the best case, $8500 in the base case, and $11 600 in the worst case.

Cost-effectiveness analysis identified a preferred screening strategy from among 13 possibilities—3 test sensitivities (70%, 80%, and 90%) and 4 frequencies (1, 2, 3, and 7 times per week) in addition to symptom-based screening—under each epidemic scenario (base, worst, and best cases) already described. We also considered the more restricted case, in which the only available test cost $25 and had a sensitivity of 80%. Finally, to help decision-makers understand the fiscal consequences of pursuing these preferred strategies, we conducted a budget impact assessment, reporting the cumulative costs for the semester on a per-student basis.

### Statistical Analysis

The model was implemented as a spreadsheet. All analyses were conducted in Microsoft Excel. Because no statistical tests were run, no prespecified level of statistical significance was set.

## Results

### Test Frequency and Sensitivity

**Figure 1.**
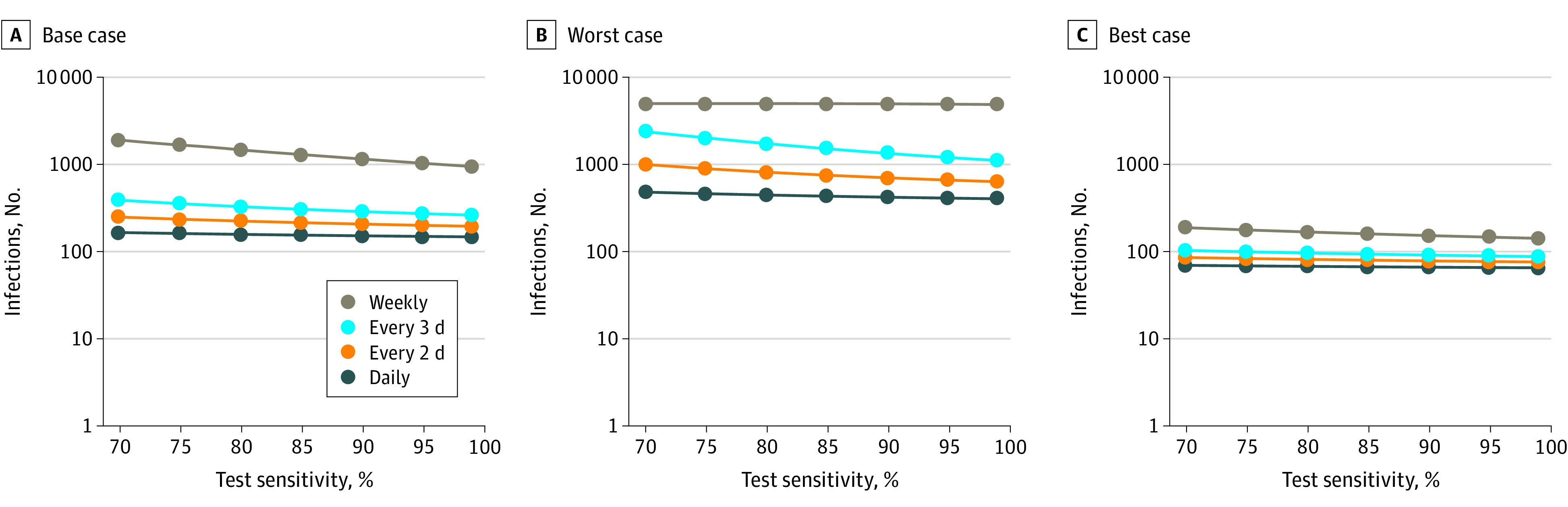
Cumulative Infections as a Function of Test Sensitivity and Frequency During an 80-day horizon, for the base case (R_t_ of 2.5, test specificity of 98%, and 10 exogenous infections per week) (A), worst case (R_t_ of 3.5, test specificity of 98%, and 25 exogenous infections per week) (B), and best case (R_t_ of 1.5, test specificity of 99.7%, and 5 exogenous infections per week) (C), these panels report cumulative infections for tests with sensitivity ranging from 70% to 99%.

At the start of the semester, the hypothetical cohort of 5000 students included 4990 (99.8%) with no SARS-CoV-2 infection and 10 (0.2%) with SARS-CoV-2 infection. During an 80-day semester in the base case (ie, R_t_ of 2.5 and 10 exogenous infections each week), screening every 1, 2, 3, or 7 days with a 70% sensitive, 98% specific test resulted in 162, 243, 379, and 1840 cumulative infections, respectively. Symptom-based screening yielded 4970 infections. Raising the sensitivity of the test from 70% to 90% reduced total infections (eg, from 162 to 149 for daily screening and from 1840 to 1118 for weekly screening). Figure 1 shows cumulative infections as a function of test sensitivity and test frequency for the 3 epidemic severity scenarios.

### Isolation Dormitory Occupancy

**Figure 2.**
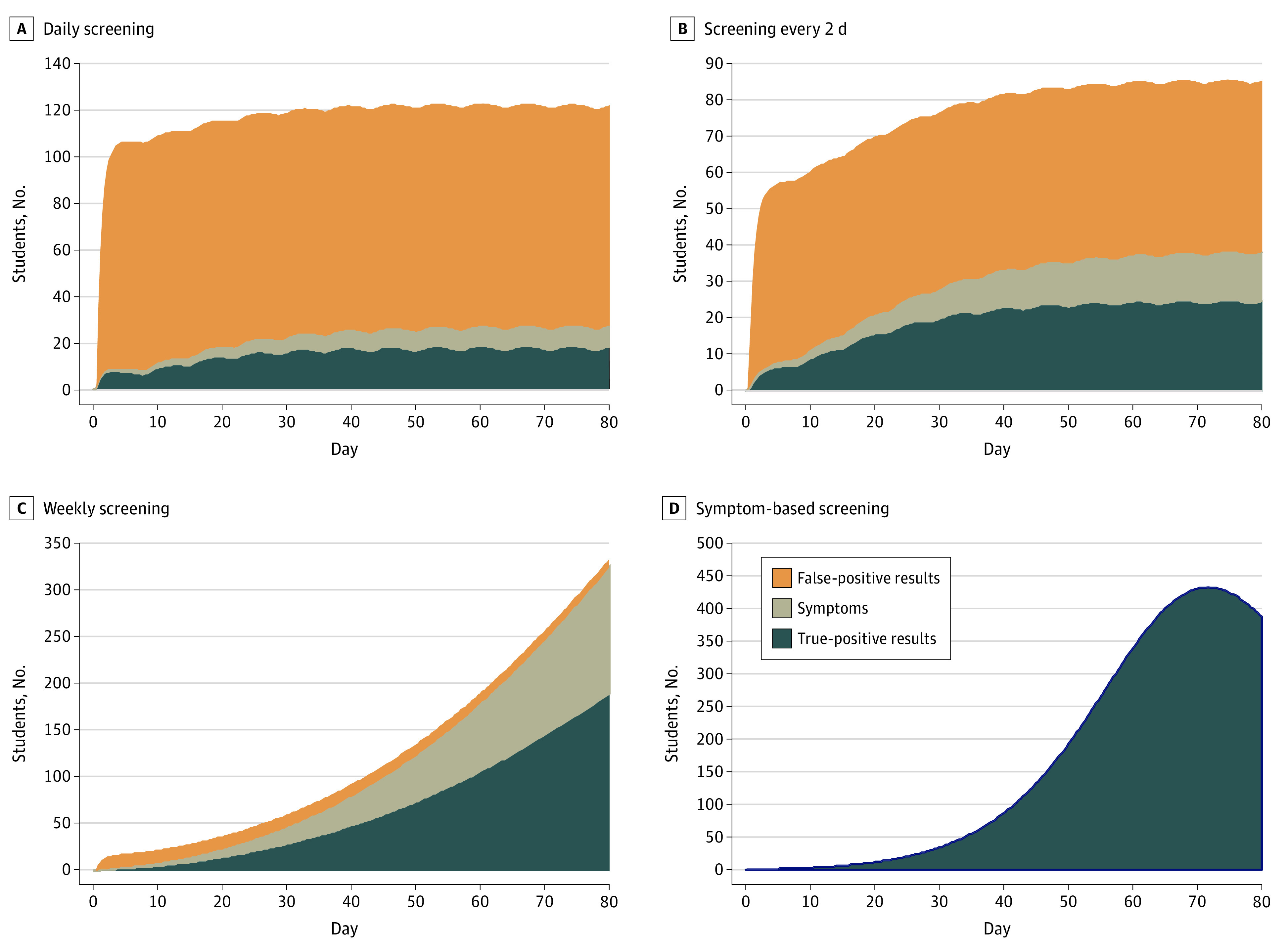
Projecting the Required Size of the Isolation Dormitory An isolation dormitory needs to be large enough to house students with false-positive results, students with symptoms, and students without symptoms who have received true-positive results. During the 80-day horizon, these panels depict the number of students in the isolation dormitory using a 70% sensitive, 98% specific test under the base case scenario (ie, R_t_ of 2.5). The effect of exogenous shocks (10 per week) is visible in the scalloped borders with daily screening and screening every 2 days (A, B); this is less evident with less frequent testing and symptom-based screening (C, D), in which the number of true-positive cases masks the comparatively small effect of exogenous shocks.

In the base case, daily screening with a 70% sensitive, 98% specific test resulted in a mean isolation dormitory census of 116 occupants, of whom 21 (18%) had true-positive results (Figure 2A). With screening every 2 days, mean daily census was reduced to 76, as fewer tests were performed and fewer false-positive results were obtained; however, less frequent testing was also associated with greater transmission of infection and a higher mean proportion of students with true-positive results in isolation (28 students [37%]) (Figure 2B). Weekly and symptom-based screening were associated with large increases in the infected occupancy of the isolation dormitory (Figure 2C and Figure 2D). For example, screening every 7 days resulted in a mean daily isolation census of 121 students, with 108 (90%) with true-positive results. Sensitivity analysis revealed that the trends evident in Figure 2 extended beyond the 80-day planning horizon (data not shown). Varying the initial number of asymptomatic infections between 0 and 100 did not materially change our findings.

The number of students with false-positive results and the isolation capacity required to accommodate them were reduced in the presence of a more specific test. For example, with daily screening in the base case, increasing the test specificity from 98% to 99.7% was associated with a decrease in the mean daily census of students with false-positive results in isolation from 95 to 15.

Under worst-case assumptions (ie, R_t_ of 3.5 with 25 exogenous infections every week), daily screening yielded mean isolation dormitory census of 152 students, of whom 60 (39%) had true infections (eFigure 2A in the Supplement). Screening every 2 days produced similar census (151) but a higher proportion (106 [70%]) of true infections (eFigure 2B in the Supplement). With weekly screening or symptom-based screening, nearly the entire student population would be infected before the conclusion of the 80-day semester (eFigure 2C and eFigure 2D in the Supplement).

In the best case (ie, R_t_ of 1.5 with 5 exogenous shocks each week and a test with 99.7% specificity), mean occupancy of the isolation dormitory was 18 (16 with infection; 2 with false-positive results) with weekly screening and 24 (all true infections) with symptom-based screening (eFigure 3 in the Supplement).

### Cost-effectiveness and Budget Impact Assessment

In the base case, screening with a less expensive, less sensitive test dominated screening with more expensive, more accurate tests (ie, it cost less and averted greater numbers of infection) for all plausible WTP values. At the benchmark maximum WTP ($8500 per infection averted), screening every 2 days with a 70% sensitive test was the preferred strategy. For WTP exceeding $28 400 per infection averted, daily screening with this same test was optimal (Table 2). Under worst-case assumptions, daily screening strategies were the only undominated choices for WTP values exceeding $4400 per infection averted; at the benchmark maximum WTP ($11 600 per infection averted), daily screening with the least sensitive (ie, 70%) test was the preferred choice. Under best-case assumptions (with a WTP maximum of $5500 per infection averted), weekly screening with a 70% sensitive test was optimal. If the only available test cost $25 and had a sensitivity of 80%, the optimal frequency of screening would be every 7, 3, and 2 days in the best, base, and worst case scenarios, respectively (eAppendix and eTable 1 in the Supplement). If the probability of progressing from infection to symptoms rose from 30% to 65%, screening every day would be optimal in the base case scenario (eTable 2 in the Supplement). During the 80-day semester, the per-student costs of implementing the preferred screening strategy were $120, $470, and $910 in the best, base, and worst case scenarios, respectively (Table 3).

**Table 2.** Results of the Incremental Cost-effectiveness Analysis in the Base-Case, Worst-Case, and Best-Case Scenarios

Frequency	Test sensitivity, %	Cost, $	Total infections	Incremental cost-effectiveness ratio, $/infection averted^a^
**Base-case scenario**^b^				
Symptom-based screening	NA	NA	4970	NA
Weekly	70	696 000	1840	200
Weekly	80	1 490 700	1422	Dominated
Every 3 d	70	1 564 500	379	600
Every 2 d	70	2 340 600	243	5700
Weekly	90	2 837 500	1118	Dominated
Every 3 d	80	3 501 800	319	Dominated
Daily	70	4 642 700	162	28 400
Every 2 d	80	5 254 900	219	Dominated
Every 3 d	90	6 740 400	280	Dominated
Every 2 d	90	10 118 700	202	Dominated
Daily	80	10 440 000	154	752 600
Daily	90	20 106 900	149	1 692 900
**Worst-case scenario**^c^				
Symptom-based screening	NA	NA	4991	NA
Weekly	70	673 600	4991	Dominated
Weekly	80	1 274 200	4988	Dominated
Every 3 d	70	1 509 300	2373	Dominated
Every 2 d	70	2 266 400	998	600
Weekly	90	2 310 000	4951	Dominated
Every 3 d	80	3 292 800	1731	Dominated
Daily	70	4 543 900	481	4400
Every 2 d	80	5 063 200	814	Dominated
Every 3 d	90	6 347 900	1335	Dominated
Every 2 d	90	9 764 100	701	Dominated
Daily	80	10 207 500	445	159 700
Daily	90	19 666 200	420	377 500
**Best-case scenario**^d^				
Symptom-based screening	NA	NA	1067	NA
Weekly	70	587 800	188	700
Every 3 d	70	1 364 600	103	9100
Weekly	80	1 432 700	168	Dominated
Every 2 d	70	2 044 500	85	38 800
Weekly	90	2 842 200	152	Dominated
Every 3 d	80	3 343 100	96	Dominated
Daily	70	4 080 900	69	128 100
Every 2 d	80	5 013 900	81	Dominated
Every 3 d	90	6 642 100	91	Dominated
Every 2 d	90	9 964 200	78	Dominated
Daily	80	10 016 800	68	3 156 700
Daily	90	19 911 200	66	6 833 800

Abbreviations: NA, not applicable.

^a^ Strategies that cost more and result in more infections than some combination of other strategies are labeled *dominated*.

^b^ Base-case scenario had a reproduction number of 2.5, 10 exogenous shock infections each week, and a maximum willingness-to-pay threshold of $8500 per infection averted.

^c^ Worst-case scenario had a reproduction number of 3.5, 25 exogenous shock infections each week, and a maximum willingness-to-pay threshold of $11 600 per infection averted.

^d^ Best-case scenario had a reproduction number of 1.5, 5 exogenous shock infections each week, a test with 99.7% specificity, and a maximum willingness-to-pay threshold of $5500 per infection averted.

**Table 3.** Per-Student Costs for Optimal Policies During an 80-Day Horizon Under Base-Case, Worst-Case, and Best-Case Scenarios

Scenario	Optimal policy	Cost per student, $
Base case, ie, R_t_ of 2.5	Screening every 2 d, 70% sensitivity	470
Worst case, ie, R_t_ of 3.5	Daily screening, 70% sensitivity	910
Best case, ie, R_t_ of 1.5	Weekly screening, 70% sensitivity	120

Abbreviation: R_t_, reproduction number.

## Discussion

The safe return of students to residential colleges demands an effective SARS-CoV-2 monitoring strategy. Results from this modelling study suggest that a highly specific screening test that can easily be administered to each student every 1 to 7 days—and that reports results quickly enough to permit newly detected cases to be isolated within hours—would be required to blunt the further transmission of infection and to control outbreaks at a justifiable cost. We identified no circumstance in this modelling study under which symptom-based screening alone would be sufficient to contain an outbreak.

Of the many uncertain variables driving our assessment of the required frequency of screening, we highlight R_t_. This uncertain measure of the transmission potential of infection will depend in part on factors that are within the control of students and university administrators. Strict adherence to handwashing, mandated indoor masking, elimination of buffet dining, limited bathroom sharing with frequent cleaning, dedensifying campuses and classrooms, and other best practices could reduce R_t_ to best-case levels, rendering containment possible with weekly testing. However, any relaxation of these measures in the residential college setting could easily increase R_t_ to worst-case levels, requiring daily screening. All members of the university community must understand the fragility of the situation and the ease with which inattention to behavior may propagate infections and precipitate the need once again to shut down campus.

Much depends on the judicious management of positive test results, both true and false. Rapid detection, confirmation, isolation, and treatment of true-positive cases is, of course, essential. We found that frequent screening with a test of modest sensitivity and a turnaround time of 8 hours would be required for this purpose. The greater difficulty lies in managing the overwhelming number of false-positives that will inevitably result from repeated screening for low-prevalence conditions. False-positive results threaten to overwhelm isolation housing capacity, a danger whose gravity increases with screening frequency. The specificity of the initial test will matter far more than its sensitivity. Many current virologic tests report a 99.8% to 100% specificity in the context of use to date for symptomatic testing^23^; we examined a value of 99.7% in the best case but used a lower value of 98% in the base-case and worst-case scenarios, given that most virologic tests have yet to be used for the kind of large-scale surveillance described in this model.

Even with a 98% specific screening test, false-positive results will present a challenge. Until a confirmatory test result is obtained, anyone receiving a positive test result will be presumed to be infectious and need to be separated from other students. Setting aside the logistic challenges and financial costs, administrators must anticipate the anxiety such separations may provoke among both students and their families. Excessive numbers of false-positive results may fuel panic and undermine confidence in the reliability of the monitoring program. It may be possible to work with test manufacturers to tune test kits under development for use in this setting, sacrificing some small measure of sensitivity in favor of higher specificity.

Obtaining an adequate supply of testing equipment will be a challenge. On a college campus with 5000 enrollees, screening students alone every 2 days will require more than 195 000 test kits during the abbreviated semester. Our analysis assumed per-test costs (including equipment and associated personnel costs) ranging from $10 to $50. Lower-cost, self-administered testing modalities may soon be available and could make screening more affordable. Pooling could also facilitate more efficient, higher-volume screening.^24^ However, pooling introduces its own logistic challenges and could increase the time to definitively identifying and isolating a positive case, resulting in further transmission and provoking anxiety among the many uninfected students notified that they are among the members of an initially positive pool.

We have tried to help decision-makers make sense of the value question by conducting a cost-effectiveness analysis and by comparing our findings with a rough estimate of the societal WTP per infection averted.^25^ While we have adhered to the broad outlines of recommended practice for the conduct of economic evaluations,^25^ we urge readers to interpret our results with caution. Most of our assumptions are conservative, ie, they understate the value of more frequent testing. For example, we ignored the clinical harms and attributable costs of COVID-19–related morbidity and treatment. We also ignored the value of infections averted beyond the student population. However, a few assumptions (eg, our failure to account for the economic and quality-of-life effects of false-positive results) may pull in the direction of less testing.

Reopening college campuses imposes risks that extend beyond students to the faculty who teach them, the many university employees (administrative and facilities staff) who come into close daily contact with them, and the countless other members of the surrounding community with whom students come into contact. University presidents have a duty to consider the downstream effect of their reopening decisions on these constituencies. However, their first responsibility is to the safety of the students in their care. While we certainly do not intend to minimize the broader effects of the reopening decision, we have quite deliberately excluded from consideration any transmissions exported off campus.

### Limitations

The simple model underlying this analysis has notable limitations. We assumed homogenous mixing without age-dependent transmission. We did not explicitly include the effect of screening on faculty and staff, although these and other nonstudent members of the college community include a higher proportion of older, more medically vulnerable individuals. We assumed that no students arrive on campus with immunity to COVID-19. We excluded the effects of contact tracing. Given its implementation challenges, this is a noteworthy omission. However, our results suggest that with frequent enough screening, contact tracing would not be necessary for epidemic control. While this analysis offers guidance on the frequency of screening, it does not speak to the logistic challenges of deploying testing strategies on large college campuses. Such challenges include the acquisition of supplies; the orchestration of screening at scale; the monitoring of adherence; the development of a strategy for rapid result return, contact, and isolation; and the availability and maintenance of an isolation dormitory with all single rooms and bathrooms.

## Conclusions

We believe that there is a safe way for students to return to college in fall 2020. In this study, screening every 2 days using a rapid, inexpensive, and even poorly sensitive (>70%) test, coupled with strict interventions that keep R_t_ less than 2.5, was estimated to yield a modest number of containable infections and to be cost-effective. This sets a very high bar—logistically, financially, and behaviorally—that may be beyond the reach of many university administrators and the students in their care.

## Notes

Supplement. **eAppendix. **Model Description **eTable 1. **Results of the Incremental Cost-effectiveness Analysis in the Base-Case, Worst-Case, and Best-Case Scenarios With a $25 Test at 80% Sensitivity **eTable 2. **Results of the Incremental Cost-effectiveness Analysis in the Base-Case Scenario With Probability of Symptoms at 65% **eFigure 1. **Model Schematic and Input Parameters **eFigure 2. **Expected Daily Occupancy of the Isolation Dormitory Under Worst-Case Assumptions **eFigure 3. **Expected Daily Occupancy of Isolation Dormitory Under Best-Case Assumptions
