# Supplementary material for: Assessment of SARS-CoV-2 Screening Strategies to Permit the Safe Reopening of College Campuses in the United States
Source: JAMA Netw Open. 2020 Jul 31;3(7):e2016818. doi: 10.1001/jamanetworkopen.2020.16818 (PMC7395236; doi:10.1001/jamanetworkopen.2020.16818)
Supplement: Supplement. — eAppendix. Model Description eTable 1. Results of the Incremental Cost-effectiveness Analysis in the Base-Case, Worst-Case, and Best-Case Scenarios With a $25 Test at 80% Sensitivity eTable 2. Results of the Incremental Cost-effectiveness Analysis in the Base-Case Scenario With Probability of Symptoms at 65% eFigure 1. Model Schematic and Input Parameters eFigure 2. Expected Daily Occupancy of the Isolation Dormitory Under Worst-Case Assumptions eFigure 3. Expected Daily Occupancy of Isolation Dormitory Under Best-Case Assumptions [file jamanetwopen-3-e2016818-s001.pdf]

Supplementary Online Content

Paltiel AD, Zheng A, Walensky RP. Assessment of SARS-CoV-2 screening strategies to permit the safe reopening of college campuses in the United States. *JAMA Netw Open*. 2020;3(7):e2016818. doi:10.1001/jamanetworkopen.2020.16818

eAppendix. Model Description
eTable 1. Results of the Incremental Cost-effectiveness Analysis in the Base-Case, Worst-Case, and Best-Case Scenarios With a $25 Test at 80% Sensitivity
eTable 2. Results of the Incremental Cost-effectiveness Analysis in the Base-Case Scenario With Probability of Symptoms at 65%
eFigure 1. Model Schematic and Input Parameters
eFigure 2. Expected Daily Occupancy of the Isolation Dormitory Under Worst-Case Assumptions
eFigure 3. Expected Daily Occupancy of Isolation Dormitory Under Best-Case Assumptions

This supplementary material has been provided by the authors to give readers additional information about their work.

© 2020 Paltiel AD et al. *JAMA Network Open.*

eAppendix. Model Description

We developed a dynamic, compartmental model using a modified "susceptible-exposed-infected-recovered" (or SEIR) framework. The model portrays the epidemiology and natural history of infection in a homogeneous population of at-risk individuals as a sequence of transitions, governed by difference equations, between different health states (or "compartments"). The flow diagram (**Figure S1**, below) illustrates the modifications we made to the basic SIR framework:

- Addition of regular, repeated screening with a test of imperfect sensitivity and specificity.
- Removal of infected individuals from the transmitting population based on either screening test findings or the development of COVID-defining symptoms.
- Removal (and return) of uninfected individuals from the transmitting population based on "false positive" screening test findings.
- Importation of additional new infections from exogenous sources (e.g., infections transmitted to students by university employees or members of the surrounding community.

Compartments. We defined a total of 8 model compartments, divided into three pools:

- <u>Active transmission and testing pool</u>. Everyone is in this pool at time 0. All transmission of infection takes place between individuals in this pool. This is also the pool in which screening for infection takes place.
    - U: Uninfected, susceptible individuals
    - E: Exposed, asymptomatic, non-infectious
    - A: Infected, asymptomatic

  Individuals in the Exposed compartment are assumed to be neither infectious nor symptomatic. (We also assume that these individuals will invariably test negative, if screened.)

  Note that, without testing, individuals in these three compartments are indistinguishable from one another.

- <u>Isolation pool</u>. Individuals in this pool are assumed to be isolated from the active transmission pool and from one another. It is assumed that transmission is not possible within this pool.
    - S: Infected, symptomatic (true) positive test result
    - TP: Infected, asymptomatic, (true) positive test result
    - FP: Uninfected, false positive result
- <u>Removed pool</u>. Individuals in this pool are presumed to be immune for the duration of the 80-day semester and therefore assumed to play no role either in the transmission of infection or in testing activities. They are removed from the active population and do not figure in the transmission equations that follow.
    - R: Recovered
    - D: Dead

© 2020 Paltiel AD et al. *JAMA Network Open.*

Parameters

β: rate at which infected individuals contact susceptibles and infect them

τ: rate at which individuals in the testing pool are screened for infection

θ: rate at which exposed individuals advance to the asymptomatic, infectious compartment

δ: rate at which individuals in the symptomatic compartment die

ρ: rate at which infected individuals recover from disease and are removed

σ: rate of symptom onset for infected individuals

μ: rate at which false positives are returned to the Uninfected compartment

Se: sensitivity of the screening test

Sp: specificity of the screening test

I(t): an indicator function which assumes value 1 if an exogenous shock takes place in cycle t; 0 otherwise

X: number of imported infections in a given exogenous shock

The model uses a cycle time of 8 hours. All rates are calculated per 8-hour cycle.

Testing is implemented in the model as a constant rate, governed by parameter τ. This means that students are screened, at random, an average once every 1/τ cycles. This does not reflect the possibility of pulsed or scheduled screening at regular intervals. Note that there is a lag of one cycle between the time that a test is conducted and the time that a student receiving a positive test result is moved to isolation; the model is specifically designed to capture this delay. This captures the time to transport the sample to the lab, obtain the result, locate the student, and effect the transfer to isolation.

© 2020 Paltiel AD et al. *JAMA Network Open.*

Governing equations

- **Uninfected (t+1) = Uninfected (t) – New Infections – New FPs + Returning FPs – Exogenous Shocks**

$$U(t+1) = U(t) \cdot \left[1 - \beta \frac{A(t)}{U(t) + A(t)}\right] - U(t-1) \cdot \tau \cdot (1 - Sp) + \mu FP(t) - X \cdot I(t+1)$$

- **Exposed (t+1) = Exposed (t) – New Infectious + New Exposeds + Exogenous Shocks**

$$E(t+1) = E(t) \cdot (1 - \theta) + \left[\beta \frac{A(t) \cdot U(t)}{U(t) + E(t) + A(t)}\right] + X \cdot I(t+1)$$

- **Asymptomatic (t+1) = Asymptomatic (t) –symptoms - recoveries+ New Infections – TPs + Exogenous Shocks**

$$A(t+1) = A(t) \cdot \left[1 - \sigma - \rho + \beta \frac{U(t)}{U(t) + A(t)}\right] - A(t-1) \cdot \tau \cdot Se + E(t) \cdot \theta$$

- **False Positives (t+1) = False Positives (t) – Returning FP+ New FPs**

$$FP(t+1) = FP(t) \cdot [1 - \mu] + U(t-1) \cdot \tau \cdot (1 - Sp)$$

- **True Positives (t+1) = True Positives (t) – Symptoms – Recovery + New TPs**

$$TP(t+1) = TP(t) \cdot [1 - \sigma - \rho] + A(t-1) \cdot \tau \cdot Se$$

- **Symptomatic (t+1) = Symptomatic (t) – Recovery – Mortality + New Symptoms**

$$S(t+1) = S(t) \cdot [1 - \rho - \delta] + \sigma[TP(t) + A(t)]$$

- **Recovered (t+1) = Recovered (t) + New Recoveries**

$$R(t+1) = R(t) + \rho\,[TP(t) + A(t) + S(t)]$$

- **Deaths (t+1) = Deaths (t) + New Deaths**

$$D(t+1) = D(t) + \delta S(t)$$

- **N = U + A + S + TP + FP + R + D = Total population size (constant)**

© 2020 Paltiel AD et al. *JAMA Network Open.*

Initial conditions:

U(0) = 4,990
A(0) = 10

All other compartments are empty at time 0.

Estimating Key Rate Parameters

1) σ: rate of symptom onset for infected individuals. We assumed that 30% of all infected individuals would eventually develop symptoms. In the absence of a screening program, this implies that $\sigma / (\sigma+\rho) = 0.3$. Assuming a mean recovery time of 14 days and computing all rates per 8-hour cycle yields $\rho = 1/(3 * 14 \text{ days})$ and we solve for $\sigma = 0.0102$.

2) β: rate at which infected individuals contact susceptibles and infect them. The effective reproductive number $Rt = \beta / (\sigma+\rho)$. We assumed Rt = {1.5, 2.5, 3.5}, which implies β = {0.051, 0.085, 0.119}. Recall that all rates are estimated per 8-hour cycle.

3) δ: rate at which individuals in the symptomatic compartment die. We assumed that the symptomatic case fatality risk was 0.05%. This implies $[\sigma / (\sigma+\rho)]*[\delta / (\delta+\rho)] = 0.0005$ and permits us to solve for $\delta = 0.00004$.

A publicly accessible version of the model implementation is available here.

© 2020 Paltiel AD et al. *JAMA Network Open.*

eTable 1. Results of the Incremental Cost-effectiveness Analysis in the Base-Case, Worst-Case, and Best-Case Scenarios With a $25 Test at 80% Sensitivity

Preferred strategies at the maximum willingness-to-pay (WTP) threshold are shaded gray.

Frequency	Cost ($)	Total Infections	Incremental Cost-effectiveness Ratio ($/infection averted)*
**Base Case Scenario (R_t 2.5, 10 exogenous shock infections each week) Maximum willingness-to-pay = $8,500/infection averted**			
**Symptom-Based Screening**	(-)	4,970	(-)
**Weekly**	1,490,700	1,422	400
**Every 3 days**	3,501,800	319	1,800
**Every 2 days**	5,254,900	219	17,500
**Daily**	10,440,000	154	80,300
**Worst Case Scenario (R_t 3.5, 25 exogenous shock infections every week) Maximum willingness-to-pay = $11,600/infection averted**			
**Symptom-Based Screening**	(-)	4,991	(-)
**Weekly**	1,274,200	4,988	dominated
**Every 3 days**	3,292,800	1,731	1,000
**Every 2 days**	5,063,200	814	1,900
**Daily**	10,207,500	445	14,000
**Best Case Scenario (R_t 1.5, 5 exogenous shock infections each week, 99.7% specific test) Maximum willingness-to-pay = $5,500/infection averted**			
**Symptom-Based Screening**	(-)	1,067	(-)
**Weekly**	1,432,700	168	1,600
**Every 3 days**	3,343,100	96	26,700
**Every 2 days**	5,013,900	81	112,300
**Daily**	10,016,800	68	366,300

*Dominated strategies are those that cost more and result in more infections than the next least costly strategy.

eTable 2. Results of the Incremental Cost-effectiveness Analysis in the Base-Case Scenario With Probability of Symptoms at 65%

The preferred strategy at the maximum willingness-to-pay (WTP) threshold are shaded gray.

Frequency	Test Sensitivity (%)	Cost ($)	Total Infections	Incremental Cost-effectiveness Ratio ($/infection averted)*
**Base Case Scenario (R_t 2.5, 10 exogenous shock infections each week, 65% with symptoms) Maximum willingness-to-pay = $8,500/infection averted**				
**Symptom-Based Screening**	(-)	(-)	4,991	(-)
**Weekly**	70	586,200	4,990	dominated
**Weekly**	80	1,178,700	4,989	dominated
**Every 3 days**	70	1,498,800	2,402	dominated
**Weekly**	70	2,207,500	4,973	dominated
**Every 2 days**	90	2,290,500	728	500
**Every 3 days**	80	3,328,100	1,599	dominated
**Daily**	70	4,612,900	248	4,800
**Every 2 days**	80	5,152,700	537	dominated
**Every 3 days**	90	6,460,700	1,114	dominated
**Every 2 days**	90	9,957,000	430	dominated
**Daily**	80	10,381,900	222	222,800
**Daily**	90	20,011,000	204	543,000

*Dominated strategies are those that cost more and result in more infections than the next least costly strategy.

© 2020 Paltiel AD et al. *JAMA Network Open.*

eFigure 1. Model Schematic and Input Parameters

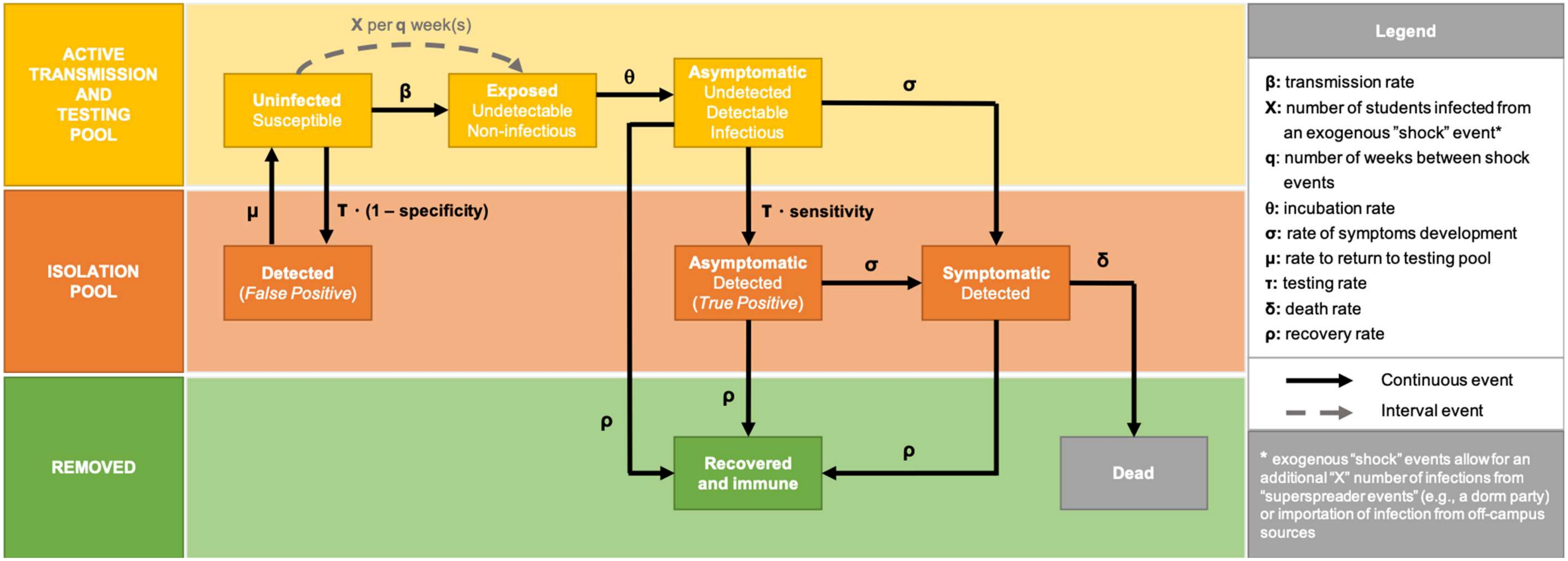

© 2020 Paltiel AD et al. *JAMA Network Open.*

eFigure 2. Expected Daily Occupancy of the Isolation Dormitory Under Worst-Case Assumptions

Worst case assumptions include R_t = 3.5, 25 exogenous shocks each week, and a 98% specific test. The panels show results of screening at different frequencies: (**a**) daily screening; (**b**) screening every 3 days; (**c**) weekly screening; and (**d**) symptom-based screening (i.e., symptom-based detection). In **Panels a and b**, the effect of exogenous shocks (25 per week) is visible in the scalloped borders; this is less evident with less frequent testing (and symptom-based screening) where the number of true positive cases masks the comparatively small impact of exogenous shocks.

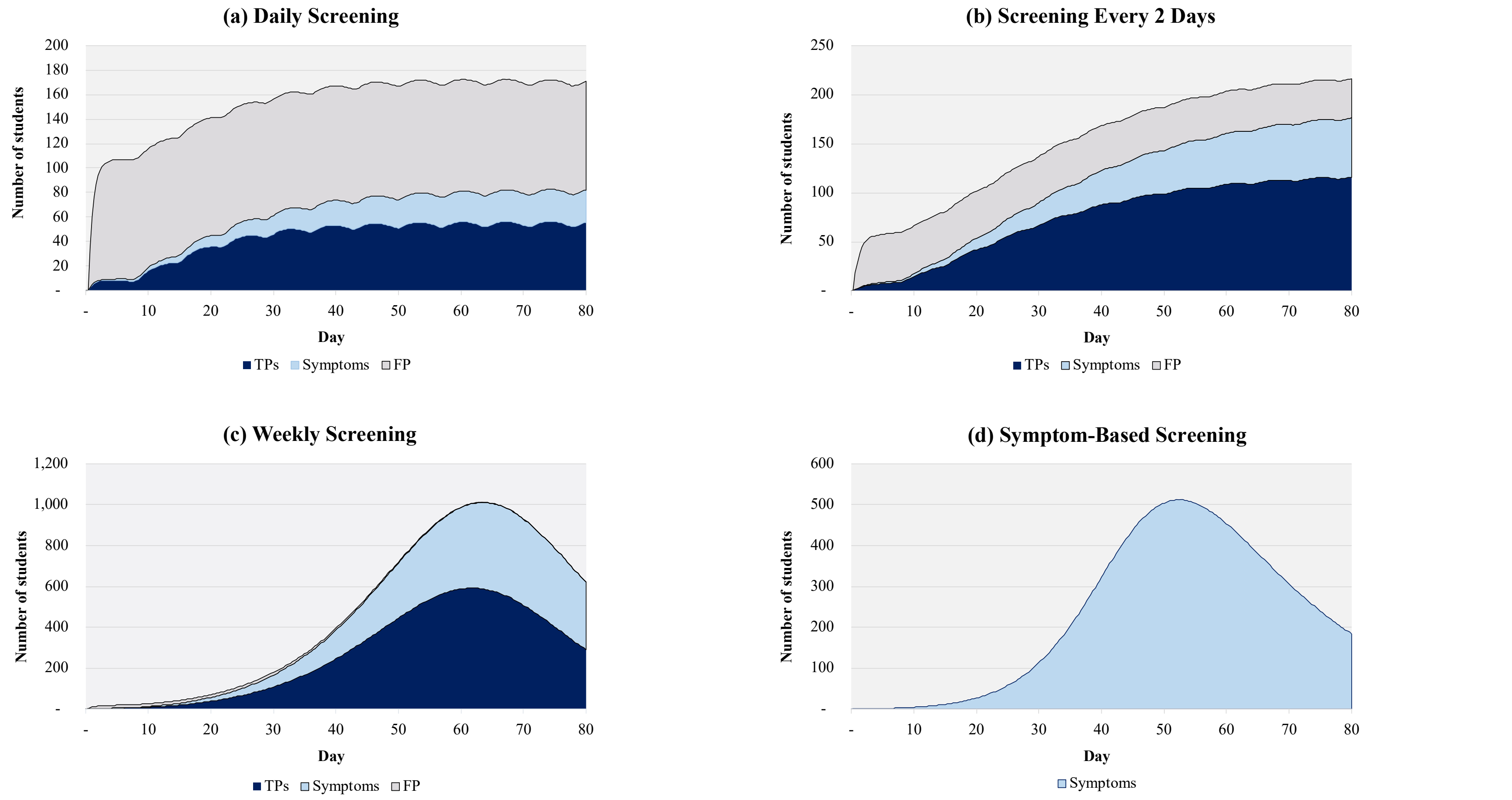

© 2020 Paltiel AD et al. *JAMA Network Open.*

eFigure 3. Expected Daily Occupancy of Isolation Dormitory Under Best-Case Assumptions
Best case assumption include R_t = 1.5, five exogenous shocks each week, and a 99.7% specific test. The panels show results of screening at different frequencies: (**a**) daily screening; (**b**) screening every 3 days; (**c**) weekly screening; and (**d**) symptom-based screening (i.e., symptom-based detection). In **Panels a and b**, the effect of exogenous shocks (5 per week) is visible in the scalloped borders; this is less evident with less frequent testing (and symptom-based screening) where the number of true positive cases masks the comparatively small impact of exogenous shocks.

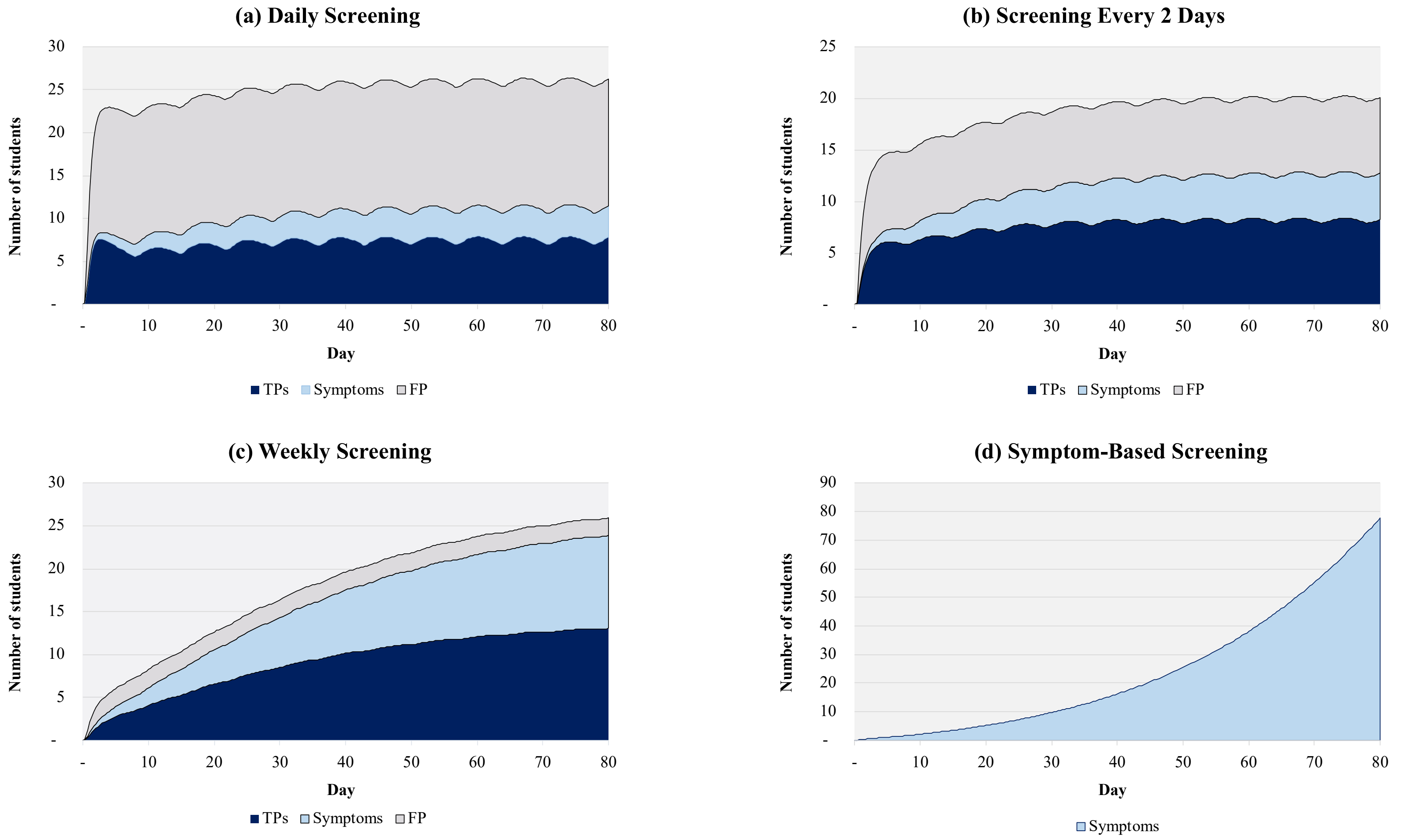

© 2020 Paltiel AD et al. *JAMA Network Open.*
